# Apocrine cystomatosis: From the aspect of epithelial-mesenchymal transition

**DOI:** 10.17221/77/2022-VETMED

**Published:** 2023-01-05

**Authors:** Tae-Un Kim, Seoung-Woo Lee, Su-Min Baek, Jae-Hyuk Yim, Young-Jin Lee, Jun-Hyeok Son, Sang-Joon Park, Jin-Kyu Park

**Affiliations:** ^1^Department of Veterinary Pathology, College of Veterinary Medicine, Kyungpook National University, Daegu, Republic of Korea; ^2^Laboratory of Veterinary Histology, College of Veterinary Medicine, Kyungpook National University, Daegu, Republic of Korea

**Keywords:** epithelial cell, cysts, EMT, morphology

## Abstract

Apocrine cystomatosis, also called epitrichial sweat gland cystomatosis, is a non-neoplastic condition characterised by multiple dilated cysts of sweat gland origin. Histopathologically, these cysts comprise two layers of cells: an inner layer of glandular epithelial cells and an outer layer of myoepithelial cells. A case of apocrine cystomatosis was admitted to a local hospital. The microscopic investigation revealed that some enlarged cysts showed the transition of glandular epithelial cells into a spindle, mesenchymal cell-like morphology. The epithelial-to-mesenchymal transition (EMT) has long been studied as a pathway for embryogenesis, organ development, and carcinogenesis. While various molecular factors, including cytokines and growth factors, are known to induce EMT, mechanical forces have also been proposed to initiate EMT. The present case describes a possible relationship between EMT occurring in a cystic condition and further pathological inspection.

Apocrine cystomatosis is an uncommon non-neoplastic disorder occurring in middle-aged or older dogs, which is characterised by solitary or multifocal cystic dilation of the epitrichial sweat glands. Cysts are usually localised within the head and neck and are characterised by a group of vesicles measuring up to 3 cm in diameter containing clear, acellular fluid. No sex and breed predisposition have been reported along with its aetiology (Stazi et al. 2022). Histopathologically, this condition is characterised by clusters of dilated apocrine glands lined by a layer of cuboidal epithelial cells surrounded by myoepithelial cells (Miller et al. 2012). Apocrine cystomatosis in other animals include swine cystomatosis observed during the post-mortem inspection in a slaughterhouse (Lopez-Figueroa et al. 2020). Nodules or vesicles filled with brown colloids were observed in the subcutaneous fat located in the dorsal region of the carcasses. The lesions, similar to canine cystomatosis, were lined by a single layer of epithelial cells surrounded by a layer of flat myoepithelial cells (Lopez-Figueroa et al. 2020). Feline cases of apocrine cystomatosis are mainly reported in the preauricular regions and the external ear canal, while less common cases were reported in the periocular, perioral and perirenal regions. The gross features of feline cystomatosis include bluish-black cystic skin and ear nodules, with a histopathology similar to that of canine cystomatosis (Loft et al. 2022).

The epithelial-to-mesenchymal transition (EMT) is a biological process in which polarised epithelial cells lose their characteristics and acquire mesenchymal cell phenotypes (Lai et al. 2020). EMT has been well studied in the context of embryogenesis and organ development and has recently been shown to play important roles in cancer progression and tissue fibrosis (Kalluri and Neilson 2003). As the transition progresses, epithelial cells lose their polarity, tight junctions, and cytokeratin intermediate filaments, and acquire a spindle morphology and mesenchymal markers as well as an enhanced migratory capacity and invasiveness (Williams et al. 2019).

EMTs are classified into three subtypes based on their biological settings. EMTs associated with embryogenesis and organ development are termed “type 1”, while those associated with wound healing, regeneration, and fibrosis, as well as carcinogenesis and metastasis, are termed “type 2” and “type 3”, respectively. Type 2 EMTs progress under inflammatory stress and directly intervene as fibrosis in the kidneys, liver, lungs, and intestines (Rastaldi et al. 2002). Type 3 EMTs are associated with cancer progression and lead to the acquisition of invasiveness and metastatic dissemination (Kalluri and Weinberg 2009). Several factors induce EMT, including the transforming growth factor-beta (TGF-β), wingless and Int-1 (Wnt) signalling pathway (Savagner 2001), and mitogenic growth factors (Uttamsingh et al. 2008).

The present report describes a case of EMT in apocrine cystomatosis in which enlarged cysts showed the transition of cuboidal epithelial cells into spindle cells, exhibiting both epithelial and mesenchymal markers.

## Case description

**Figure 1:**
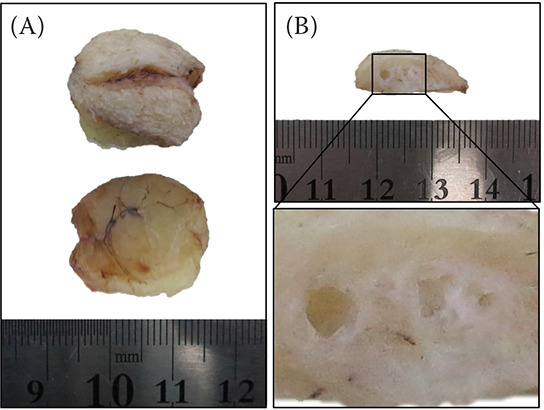
Gross findings of an inguinal nodule The nodule is 2 cm in diameter (A). The cut surface is a yellowish-white colour with accompanying cystic structures (B)

A 12-year-old spayed male Cocker Spaniel was admitted to a local animal hospital with an inguinal nodule. After the basic examination, a skin biopsy of the lesion was performed for a histopathological examination. Grossly, the nodule was 2 cm in diameter, and the cut surface exhibited a yellowish-white colour with accompanying cystic structures (Figure 1A,B).

The resected sample was fixed in 10% neutral buffered formalin and routinely processed for a histopathological diagnosis. The processed paraffin blocks were serially sectioned at a 3 μm thickness for the microscopic and immunohistochemical analyses.

Immunohistochemical labelling was performed with antibodies against pan-cytokeratin (mouse monoclonal, 1 : 200; Abcam, Cambridge, UK) as an epithelial cell marker and α-SMA (mouse monoclonal, 1 : 1 000, M0851; Dako, Glostrup, Denmark) and vimentin (mouse monoclonal, 1 : 50, MA5-11883; Invitrogen, Waltham, MA, USA) as a marker for mesenchymal cells. The sectioned tissue slides were deparaffinised in toluene and rehydrated in serially diluted ethanol (100, 95, 90, 80, and 70%) and distilled water. Antigen retrieval was performed in 3% hydrogen peroxide in methanol at room temperature for 30 min and steamed for 30 min in a 10 mmol/l citric acid buffer.

After cooling at room temperature for 2 h, the slides were washed and covered with a blocking solution (Life Technologies, Frederick, MD, USA) for an hour. Each slide was then covered with primary antibodies and incubated overnight at 4 °C. The next day, the slides were washed and thoroughly incubated with a broad-spectrum second antibody and a streptavidin-horseradish peroxidase conjugate (Life Technologies, Frederick, MD, USA) at room temperature for 10 min each. Diaminobenzidine (DAB; Vector Laboratories, Burlingame, CA, USA) was used for the visualisation. The slides were counterstained with 10% haematoxylin for 5 min and covered after dehydration.

Immunofluorescence double labelling of α-SMA (mouse monoclonal, 1 : 800, M0851; Dako, Glostrup, Denmark) and pan-cytokeratin (rabbit polyclonal, 1 : 50, ab9377; Abcam, Cambridge, UK) was performed. Double labelling of vimentin (mouse monoclonal, 1 : 50, MA5-11883; Invitrogen, Waltham, MA, USA) and pan-cytokeratin (rabbit polyclonal, 1 : 50, ab9377; Abcam, Cambridge, UK) was also performed. Slides were incubated with a 0.1% Triton X-100 solution for 20 min at room temperature for permeabilisation.

The sections were then blocked with 5% donkey serum for an hour, and incubated overnight with the primary antibodies at 4 °C. The next day, the slides were washed and treated with the secondary antibodies, Alexa Fluor^®^ 488 donkey anti-mouse IgG (1 : 500, ab150105; Abcam, Cambridge, UK) and Alexa Fluor^®^ 555 donkey anti-rabbit IgG (1 : 500, ab150067; Abcam, Cambridge, UK), for an hour at room temperature. The ProLong Gold Antifade Reagent with 4', 6-diamidino-2-phenylindole, dihydrochloride (DAPI) (Cell Signalling Technology, Danvers, MA, USA) was used for the nuclear detection.

**Figure 2:**
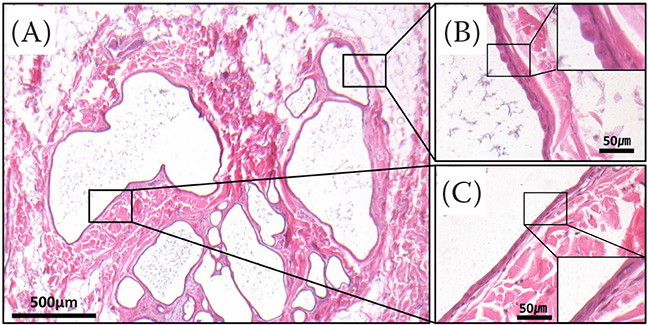
Microscopic findings; haematoxylin & eosin staining (A) Multiple cystic lesions of various sizes, × 40. (B) The cysts are lined with a layer of glandular epithelial cells surrounded by a layer of myoepithelial cells, × 400. (C) Enlarged cysts with two layers of spindle cells, × 400

**Figure 3:**
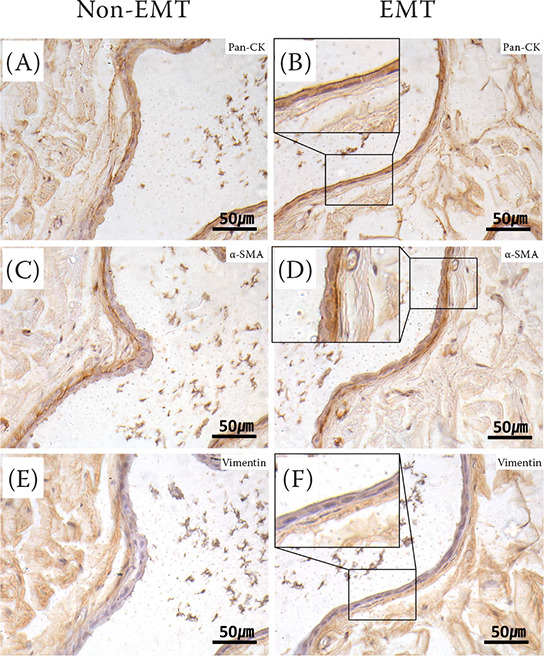
Immunohistochemistry of the cystic linings; × 400. Cuboidal (A) and spindle (B) epithelial cells positive for pan-cytokeratin, × 400. Cuboidal epithelial cells negative for α-SMA and vimentin (C,E). Epithelial cells with spindle morphology positive for α-SMA and vimentin labelling (D,F) EMT = epithelial-to-mesenchymal transition

Microscopically, the sample was composed of multiple dilated cysts (Figure 2A) lined by two layers of cells: an inner single layer of cuboidal glandular epithelial cells surrounded by an outer single layer of myoepithelial cells (Figure 2B). The glandular epithelial cells were characterised by cuboidal cells with dense nuclei and apical blebbing with no cell pleomorphism, suggesting an apocrine gland origin. No signs of excess proliferation, such as multi-layered epithelial cells or papillary growth, were observed. The immunohistochemistry revealed that the inner layer of glandular epithelial cells was positive for the pan-cytokeratin antibody (Figure 3A), while the outer layer of myoepithelial cells was positive for α-SMA (Figure 3C).

**Figure 4:**
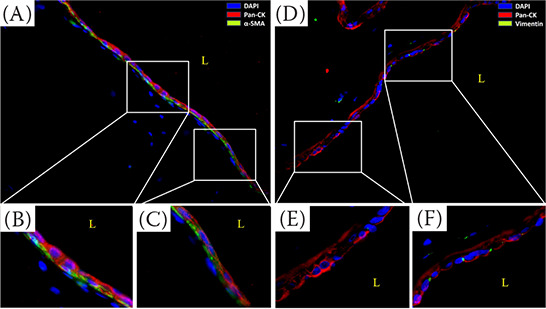
Immunofluorescent labelling of the cystic structures (A–C) Double-labelling of pan-cytokeratin and α-SMA. (B) Cuboidal epithelial cells strongly positive for pan-cytokeratin. (C) Spindle epithelial cells positive for both pan-cytokeratin and α-SMA. (D–F) Double-labelling of pan-cytokeratin and vimentin. (E) Cuboidal epithelial cells positive for pan-cytokeratin. (F) Spindle epithelial cells positive for both pan-cytokeratin and vimentin L = lumen

Unlike the cystic structures described in previous cases of cystomatosis with cuboidal epithelial cells in the inner layer, some enlarged cysts in the present case were lined with two layers of cells, both exhibiting mesenchymal cell-like morphology (Figure 2C). The immunohistochemical analysis showed that the inner layer of spindle cells, which should have been cuboidal epithelial cells, showed positive expression of pan-cytokeratin (Figure 3B), α-SMA, and vimentin (Figure 3D,F), indicating that this spindle layer had the characteristics of both epithelial and mesenchymal cells. For further investigation, immunofluorescence double labelling was performed. While the cuboidal epithelial cells showed strong single labelling of pan-cytokeratin (Figure 4C,E), the epithelial cells with the spindle morphology showed a loss of pan-cytokeratin expression, but gained α-SMA positivity (Figure 4D). A similar pattern was observed in the double labelling of pan-cytokeratin and vimentin, with vimentin positivity emerging in the perinuclear region of the pan-cytokeratin-positive epithelial cells (Figure 4F).

## DISCUSSION

The differential diagnosis of cystomatosis includes apocrine cystadenomas, which are proliferative neoplasms. Cystadenomas require epithelial cell proliferation characterised by acinar or papillary projections into the lumen. Immunohistochemistry is useful for distinguishing cystomatosis from cystadenomas, since non-neoplastic cystomatosis shows strong positivity for α-SMA in the basal layer of the lesion with a low proliferative index, whereas neoplastic cystadenomas show a higher proliferative index of epithelial cells than cystomatosis with mild to absent α-SMA (Miyamoto et al. 2005). The cysts in the present case comprised two layers of cells: an inner layer of cuboidal epithelial cells positive for pan-cytokeratin and an outer layer of myoepithelial cells positive for α-SMA. The absence of papillary growth indicated low proliferative activity, confirming the non-neoplastic nature of the lesion. Thus, the sample was diagnosed as apocrine cystomatosis originating from the sweat gland.

A study on EMT in cystic structures was reported in polycystic kidney (PCK) rat models (Togawa et al. 2011). Multiple cyst formation in the nephron segments of the PCK rats corresponded to the high expression of the E-cadherin epithelial cell adhesion protein, which decreased in parallel with the cyst enlargement. Along with the gradual loss of the E-cadherin expression, the acquisition of mesenchymal features in the epithelial cells was observed in the enlarged cysts, suggesting the significance of the EMT in the pathophysiology of cystic diseases (Togawa et al. 2011). In the present case, the cystic structures of apocrine gland origin showed cuboidal epithelial cells becoming spindle-shaped following cyst enlargement. The immunohistochemistry and immunofluorescence labelling confirmed that the spindle cells possessed both epithelial and mesenchymal features, showing evidence of EMT in the enlarged cysts.

The relationship between cyst enlargement and EMT initiation has not been confirmed. One hypothesis is that the EMTs of enlarged cysts are precursors of fibroblasts that mediate organ fibrosis. Various molecules generated by inflammatory cells or resident myoepithelial cells cause degradation of the cell-cell adherence, thereby inducing EMT (Kalluri and Weinberg 2009). However, the microscopic investigation in the present case showed no significant signs of inflammation capable of inducing fibrogenesis. Another hypothesis is that the mechanical force produced by cyst enlargement drives EMT. In recent years, researchers have proposed that extracellular force induces EMT in epithelial cells, thereby changing the cell fate (Przybyla et al. 2016). EMT initiated by mechanical force enables cells to survive without adherence, and, along with precancerous mutations or epigenetic changes, accelerates neoplastic proliferation (Frisch et al. 2013). Due to its low incidence, the correlation between non-neoplastic cystomatosis and neoplastic cystadenoma has not yet been studied. However, cyst enlargement with the existence of EMT may progress to neoplastic lesions, such as cystadenomas from pre-existing cystomatosis, suggesting that EMT may play an important role in the pathophysiology of cystic lesions.
